# Identification and characterization of extrachromosomal circular DNA in Wei and Large White pigs by high-throughput sequencing

**DOI:** 10.3389/fvets.2023.1085474

**Published:** 2023-02-03

**Authors:** Aiyou Wen, Wei Zhang, Jingen Xu, Kunping Wang, Hong Hu

**Affiliations:** ^1^College of Animal Science, Anhui Science and Technology University, Chuzhou, China; ^2^Institute of Animal Husbandry and Veterinary Medicine, Anhui Academy of Agricultural Sciences, Hefei, China

**Keywords:** eccDNA, ecDNA, Wei pig, Large White pig, high-throughput sequencing

## Abstract

Wei pig (WP) and Large White pig (LP) are fatty and lean breeds, respectively. Extrachromosomal circular DNA (eccDNA) plays an important role in regulating signaling pathway processes of cell. However, there are few reports regarding the eccDNA and ecDNA profiles in WP and LP. The present work aimed to investigate the eccDNA and ecDNA profiles between WP and LP. Three WPs and three LPs (100 ± 1.3 kg) were selected for analysis of eccDNA and ecDNA in the ear samples. Results showed that there were 39,686,953,656–58,411,217,258 and 53,824,168,657–58,311,810,737 clean data for WP and LP, respectively. Sequencing yielded 15,587–25,479 and 71,123–79,605 eccDNAs from the ear samples of WP and LP, respectively. There were 15,111 and 22,594 eccDNA-derived genes in the WP and LP, respectively, and 13,807 eccDNA-derived genes were common in the ear samples of both pigs. Sequencing yielded 13–19 and 27–43 ecDNAs in the ears of WP and LP, respectively. There were 1,005 and 1,777 ecDNA-derived genes in WP and LP, respectively, and 351 ecDNA-derived genes were common in the ear samples of both pigs. The most significant KEGG pathways of eccDNA-derived genes were axon guidance, focal adhesion, metabolic pathways, MAPK signaling pathway, Hedgehog signaling pathway, microRNAs in cancer, tight junction, phospholipase D signaling pathway, endocytosis, and sphingolipid signaling pathway. Furthermore, the most significant KEGG pathways of ecDNA-derived genes were olfactory transduction, B cell receptor signaling pathway, and chemical carcinogenesis. The eccDNA00044301 was lower abundance, while the ecDNA00000060 was higher abundance in WP compared with that in LP. Summary, we found that eccDNAs and ecDNAs are common in WP and LP and occur in sizes large enough to carry one or several partial or complete genes. These findings have expanded the knowledge repertoire of circular DNA in pig and will provide a reference for the use of pigs as a medical model and help discovery of new genetic markers to select high-quality breeds.

## Introduction

Extrachromosomal circular DNA (eccDNA), an unconventional presence of extrachromosomal DNA, is a circular DNA molecule derived from genomic DNA (1, 2). It can be categorized as microDNA, spcDNA, and ecDNA. The ecDNA which found in cancerous cell are double-stranded circular DNA molecule ranging from a few 100 kb to several Mbs in size (3, 4). It has recently been shown that eccDNA plays an important role in regulating biological processes including signal pathway, telomere trimming, and stress response (5, 6). The special distribution of eccDNA enhances the ability to use it as a biomarker for some diseases, especially cancer (7). With the development of high-throughput sequencing, eccDNA sequencing may be helpful for researchers to further explore the potential biological functions of eccDNA.

The pig is an ancient omnivorous mammal. As an economically important animal in agriculture, pigs are a main source of meat products and are in high demand worldwide (8, 9). The similarities between pig and human, in terms of genome, immunology, and, anatomical structure, enhance its usefulness as a biological model (10–12). Pigs have become an important biomedical research model for studying obesity, diabetes, hypertension, and other human diseases (13).

The Wei pig (WP) is a famous fatty breed in Anhui Province of China; it is resistant to stress and has a high reproductive rate and fat content (14). The characteristics of Large White pig (LP, an excellent lean breed) include a fast growth speed, high lean rate, and low feed gain ratio (14, 15). There are some differences between the two varieties owing to differences in their genetic backgrounds. However, few reports are available regarding the differences in eccDNA and ecDNA between WP and LP. The present work used high-throughput sequencing to determine eccDNA and ecDNA profiles from the ear samples of WP and LP. Bioinformatics was used to assess the similarities and differences of eccDNA and ecDNA between WP and LP. This information could provide a reference for the potential use of pigs as a medical model and help discover new genetic markers to select high-quality breeds.

## Materials and methods

### Experiment design

This experiment was approved by the Animal Care and Use Committee of Anhui Science and Technology University. Three male WPs and three male LPs (weight 100 ± 1.3 kg) were selected from cooperative farm of Anhui Science and Technology University were dived into CW and CY groups. The ear samples were collected from these pigs and stored at −70°C until the DNA extraction procedure.

### Library construction, sequencing, and circular DNA analysis

The DNA of ear sample was extracted use a commercial kit (Tiangen, China) and the quality was detected using Qubit (ThermoFisher, USA) and NanoDrop (ThermoFisher, USA). The library construction and sequencing were carried out according to the methods described by Møller et al. (16). The samples were processed by exonuclease V (NEB, USA) to degrade the linear genomic DNA For the restriction enzyme-based approach, circular DNA were digested using MspI (NEB, USA). Then, the library was sequenced on Novaseq 6000 (Illumina, USA) and 150 paired-end reads were generated (GeneDenovo, China). The high-quality clean reads were obtained from raw reads by Ffastp software. Q20 and Q30 were used to determine the quality of circular DNA. Large circular DNA in tumors is generally > 100 kb, while the other types of circular DNA are usually below 100 kb. Therefore, we used 100 kb as a threshold to distinguish eccDNA from ecDNA. The corresponding correlation analysis was performed independently.

### Analysis of cDNA distribution and its derived genes

EccDNA and ecDNA were annotated according to its source region. We divided the genome into exons, introns, gene_up2k, gene_down2k, and intergenic regions and annotated each eccDNA and ecDNA based on the region to which it belonged. If the eccDNA and ecDNA spanned multiple regions, it was classified according to the following priority: exon > intron > gene_ up2k > gene_ down2k > intergenic. The eccDNA and ecDNA related genes were derived from coding gene regions. The common and unique eccDNA and ecDNA related gene between CW and CY groups were analyzed by Venn analysis (https://www.omicshare.com).

### Differential abundance analysis of eccDNA and ecDNA

The differential abundance of eccDNA and ecDNA were analyzed using EdgeR software. The eccDNA and ecDNA with a *p*-value < 0.05 and a |log_2_FC| > 1 was regard as the significant difference between CW and CY groups.

### Analysis of GO and KEGG

The enriched pathway of eccDNA and ecDNA related genes were performed using GO and KEGG database in www.omicshare.com.

## Results

### Summary of high-throughput sequencing

Sequencing yielded 39,686,953,656–58,411,217,258 and 53,824,168,657–58,311,810,737 clean data for WP and LP, respectively (Table 1). The average Q30 values for WP and LP were 93.69 and 92.4%, respectively. An average of 80.46% (WP) and 81.44% (LP) of reads were mapped along the *Sus scrofa* reference genome (Table 1). The average GC content of WP and LP was 49.78 and 54%, respectively.

**Table 1:** Summary of high-throughput sequencing.

**Pig**	**Raw data (bp)**	**Clean data (bp)**	**Q20 (%)**	**Q30 (%)**	**Total mapped (%)**	**GC (%)**
CW1	72,057,443,400	58,301,151,414	97.50%	93.54%	80.51	48.22
CW2	67,214,298,000	58,411,217,258	97.24%	93.46%	79.79	50.90
CW3	47,459,279,100	39,686,953,656	97.62%	94.04%	81.08	50.23
CY1	58,233,148,200	54,898,548,653	96.81%	92.54%	79.66	52.40
CY2	58,510,024,200	53,824,168,657	96.42%	92.18%	79.91	56.80
CY3	61,656,094,500	58,311,810,737	96.86%	92.48%	84.75	52.79

CW, Wei pig; CY, Large White pig.

### Identification of eccDNA and its derived genes

**Figure 1:**
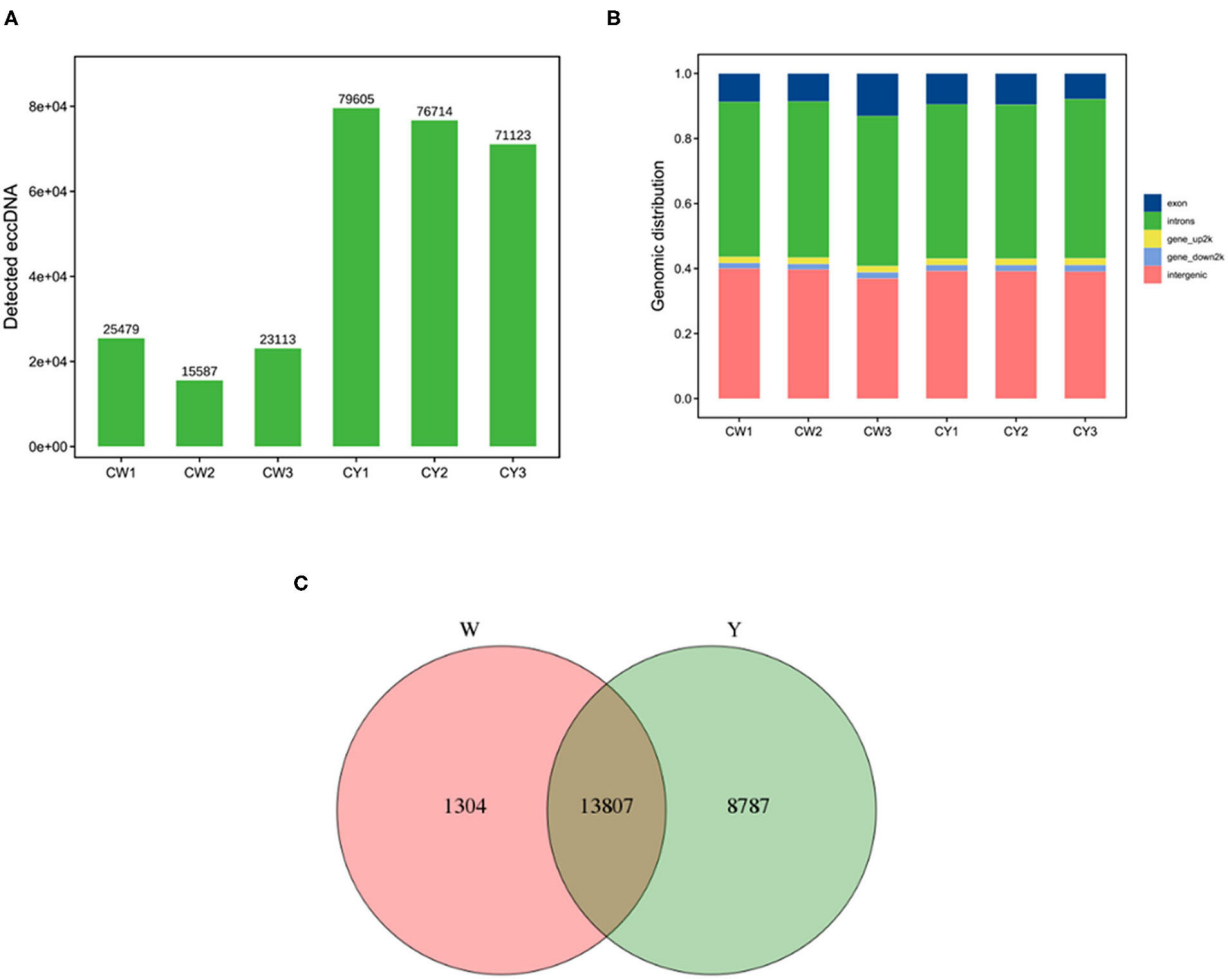
Identification of eccDNA and its derived genes. **(A)** Identification of eccDNA in Wei (CW) and Large White (CY) pigs. **(B)** Distribution of eccDNA. **(C)** Venn analysis of eccDNA derived genes in Wei and Large White pigs.

As shown in Figure 1A, sequencing yielded 15,587–25,479 and 71,123–79,605 eccDNAs in the ears of WP and LP, respectively. Most eccDNAs belonged to introns and intergenic regions (Figure 1B). There were 15,111 and 22,594 eccDNA-derived genes in the WP and LP, respectively, and 13,807 eccDNA-derived genes were common in the ears of both pig types (Figure 1C).

### Identification of ecDNA and its derived genes

**Figure 2:**
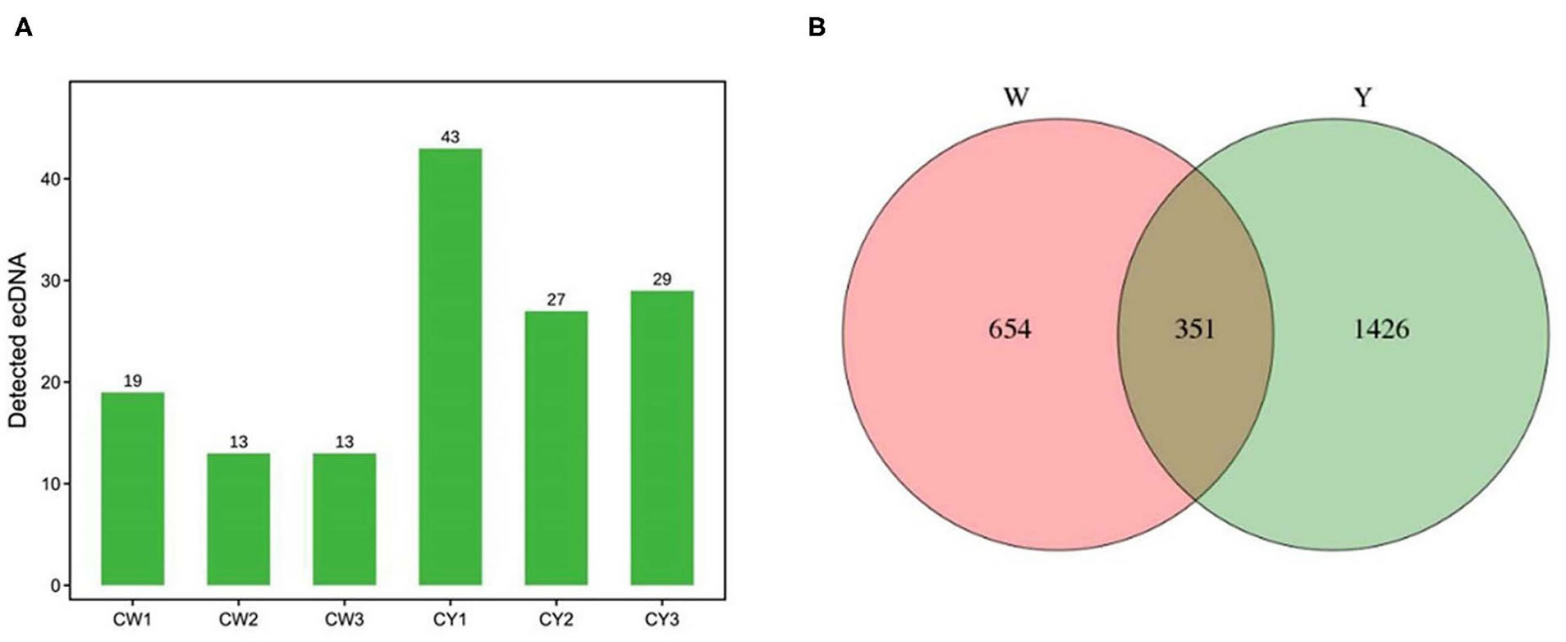
Identification of ecDNA and its derived genes. **(A)** Identification of ecDNA in Wei (CW) and Large White (CY) pigs. **(B)** Venn analysis of ecDNA derived genes in Wei and Large White pigs.

Figure 2A shows that sequencing yielded 13–19 and 27–43 ecDNAs in the ears of WP and LP, respectively. There were 1,005 and 1,777 ecDNA-derived genes in WP and LP, respectively, and 351 ecDNA-derived genes were common in the ears of both pig types (Figure 2B).

### GO and KEGG analyses of eccDNA-derived genes in WP and LP

As shown in Table 2, the GO enriched terms of eccDNA-derived genes including biological processes (cellular component organization, cellular component organization or biogenesis, metabolic process, etc.), cellular components (cell, cell part, intracellular etc.), and molecular functions (protein binding, catalytic activity, ion binding etc.) in the WP and LP.

**Table 2:** GO analysis of common eccDNA derived genes in Wei and Large White pigs.

**GO ID**	***q*-value**	**Class**	**Description**
GO:0016043	1.81E-62	Biological process	Cellular component organization
GO:0071840	8.28E-57		Cellular component organization or biogenesis
GO:0008152	1.03E-52		Metabolic process
GO:0071704	3.80E-45		Organic substance metabolic process
GO:0048856	4.21E-43		Anatomical structure development
GO:0044237	1.98E-42		Cellular metabolic process
GO:0032502	5.80E-42		Developmental process
GO:0051234	1.45E-37		Establishment of localization
GO:0007275	1.78E-37		Multicellular organism development
GO:0048518	2.32E-39		Positive regulation of biological process
GO:0005623	7.20E-272	Cellular component	Cell
GO:0044464	7.20E-272		Cell part
GO:0005622	5.30E-208		Intracellular
GO:0044424	5.30E-208		Intracellular part
GO:0043226	1.02E-146		Organelle
GO:0005488	1.08E-141		Binding
GO:0005737	1.90E-133		Cytoplasm
GO:0043229	4.12E-132		Intracellular organelle
GO:0043227	6.93E-104		Membrane-bounded organelle
GO:0044444	3.68E-92		Cytoplasmic part
GO:0005515	1.87E-106	Molecular function	Protein binding
GO:0003824	2.69E-60		Catalytic activity
GO:0043167	5.55E-58		Ion binding
GO:0043168	4.53E-48		Anion binding
GO:0032559	7.20E-44		Adenyl ribonucleotide binding
GO:0030554	1.69E-43		Adenyl nucleotide binding
GO:0005524	6.89E-42		ATP binding
GO:0008144	2.02E-40		Drug binding
GO:0000166	4.15E-40		Nucleotide binding
GO:1901265	4.15E-40		Nucleoside phosphate binding

**Figure 3:**
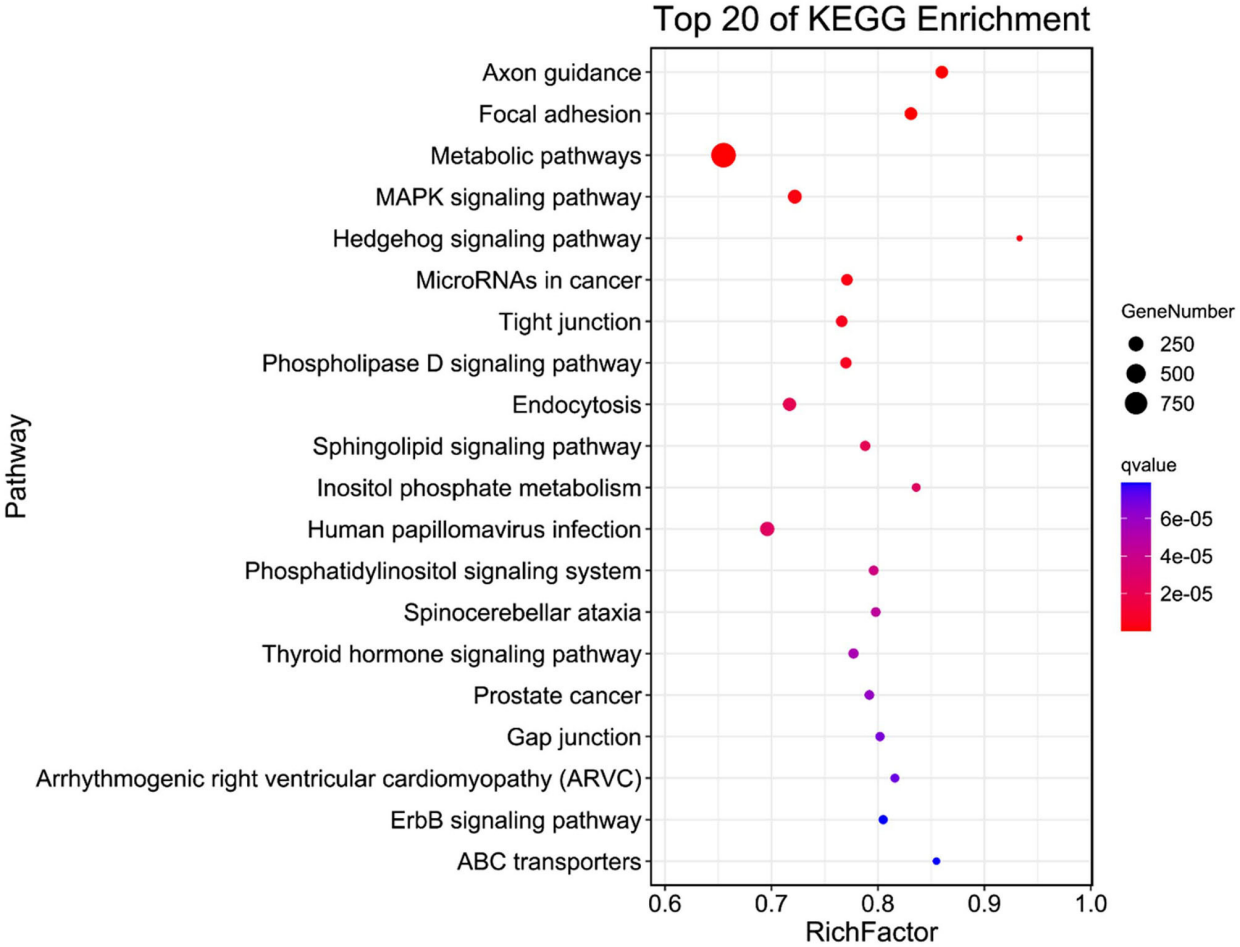
KEGG analysis of common eccDNA derived genes in Wei and Large White pigs.

Figure 3 shows the top 20 most significant KEGG pathways, including axon guidance, focal adhesion, metabolic pathways, MAPK signaling pathway, Hedgehog signaling pathway, microRNAs in cancer, tight junction, phospholipase D signaling pathway, endocytosis, and sphingolipid signaling pathway in the WP and LP.

### GO and KEGG analyses of ecDNA-derived genes in WP and LP

Table 3 shows the GO enriched terms of ecDNA-derived genes, including biological processes (G protein-coupled receptor signaling pathway), cellular components (membrane, integral component of membrane, intrinsic component of membrane, etc.), and molecular functions (olfactory receptor activity, N-acyltransferase activity, G protein-coupled receptor activity, etc.) in the WP and LP.

**Table 3:** GO analysis of common ecDNA derived genes in Wei and Large White pigs.

**GO ID**	***q*-value**	**Class**	**Description**
GO:0007186	0.004718	Biological process	G protein-coupled receptor signaling pathway
GO:0016020	0.034356	Cellular component	Membrane
GO:0016021	0.034356		Integral component of membrane
GO:0031224	0.035249		Intrinsic component of membrane
GO:0004984	3.22E-05	Molecular function	Olfactory receptor activity
GO:0016410	0.00047		N-acyltransferase activity
GO:0004930	0.00047		G protein-coupled receptor activity
GO:0004888	0.002816		Transmembrane signaling receptor activity
GO:0038023	0.003332		Signaling receptor activity
GO:0060089	0.003332		Molecular transducer activity
GO:0008080	0.003332		N-acetyltransferase activity
GO:0015019	0.006628		Heparan-alpha-glucosaminide N-acetyltransferase activity
GO:0033754	0.006628		Indoleamine 2,3-dioxygenase activity
GO:0016407	0.009387		Acetyltransferase activity
GO:0016747	0.009387		Transferase activity, transferring acyl groups other than amino-acyl groups
GO:0004060	0.013001		Arylamine N-acetyltransferase activity
GO:0008392	0.013001		Arachidonic acid epoxygenase activity
GO:0005452	0.013001		Inorganic anion exchanger activity
GO:0016746	0.018849		Transferase activity, transferring acyl groups
GO:0008391	0.022023		Arachidonic acid monooxygenase activity

As shown in Table 4, the top 3 most significant KEGG pathways including olfactory transduction, B cell receptor signaling pathway, and chemical carcinogenesis in the WP and LP.

**Table 4:** KEGG analysis of common ecDNA derived genes in Wei and Large White pigs.

**Pathway**	**Class**	**KEGG ID**	***q*-value**
Olfactory transduction	Organismal systems	ko04740	1.60E-09
B cell receptor signaling pathway	Organismal systems	ko04662	0.0141
Chemical carcinogenesis	Human diseases	ko05204	00179

### Differential abundance of eccDNA and ecDNA

As shown in Table 5, eccDNA00044301 was lower abundance in WP compared with that in LP. Furthermore, the gene derived from eccDNA00044301 is a rhomboid 5 homolog 2 (RHBDF2). The ecDNA00000060 was higher abundance in WP compared with that in LP.

**Table 5:** Differential abundance of eccDNA and ecDNA in Wei and Large White pigs.

**ID**	**Log_2_FC**	***P*-value**	**Type**	**Annotation**	**Symbol**
EccDNA00044301	−19.091	0.04913	Introns	ENSSSCG00000023362	RHBDF2
EcDNA00000060	5.64542	0.03727	Intergenic	Not available	Not available

## Discussion

Generally, most DNA is linear in eukaryote. Nevertheless, some unconventional DNAs, existed in extrachromosomal region, are found to be circular (2, 17). The extrachromosomal cDNA is highly correlated with disease occurrence (7, 18). Pigs and humans are not only highly similar in disease occurrence and physiological characteristics, but also have a high homology in genome and chromosome structure; therefore, pigs have been used as an important mammalian model in human research (19). Many studies have used high-throughput sequencing to study the differences in mRNA, microRNA, and lncRNA in different breeds of pigs, but to the best of our knowledge, there are no previous reports on the high-throughput sequencing of circular DNA in pigs (20–22). In this study, high-throughput sequencing was used to investigate their differences in circular DNA between fatty and lean pig breeds to lay a foundation for their use as human research models and in livestock breeding.

DNA is mainly located in the nucleus of the cells of humans, animals, and plants. However, in special cases, excluding exogenous nucleic acid invasions such as exogenous virus infection, some DNA particles are present outside the chromosome (23, 24). These extrachromosomal DNA particles can be linear or circular. Initially, cDNA was identified in human tumor cells and was thought to be associated with tumor heterogeneity and drug resistance (25, 26). Further studies showed that eccDNA was present in yeast, tumor samples, and cancer cell lines (27, 28). Møller et al. (16) suggested that cDNAs are found in the somatic tissue of healthy humans (16). Similar results were obtained in this study. About > 80% clean reads were mapped to *S. scrofa* genome, and many eccDNAs and ecDNAs were isolated from the ear samples of WP and LP. Most eccDNAs belonged to the intron and intergenic regions. These results demonstrated that the eccDNA and ecDNA were not only existed in the tumor cells, but also existed in the somatic tissue.

EccDNA and ecDNA usually carry partial or complete genes and functional elements, and participate in aging, drug resistance, and tumors (27, 29, 30). In the GO enrichment analysis, the eccDNA- and ecDNA-derived genes were involved in some pathways, including metabolic process, cell, cell part, olfactory receptor activity, and catalytic activity. The KEGG pathway, detected in terms of the number of genes and q value, was analyzed to further discover the potential biological functions of eccDNA and ecDNA-derived genes in the present study. The most significant KEGG pathways of eccDNA-derived genes were annotated as lipid metabolism, folding, sorting and degradation, signal transduction, transport and catabolism, endocrine system, and infectious diseases. Furthermore, the most significant KEGG pathways of ecDNA-derived genes were annotated as organismal systems and human diseases. These results suggest that eccDNA and ecDNA may be important in regulating metabolism and disease occurrence (18, 31). In particular, KEGG pathway annotation of human diseases (e.g., cancers, infectious diseases, endocrine and metabolic diseases, and immune diseases) suggested that WP and LP have significant potential to construct biomedical disease models.

To further investigate the differences in cDNA, the differential abundance of eccDNA and ecDNA was analyzed in WP and LP. EccDNA00044301 was higher abundance in LP than that in WP. The gene derived from EccDNA00044301 is RHBDF2. This gene is important for iRhoms related to physiological targets, development, disease, and targeting therapeutic opportunities (32). The iRhoms have crucial functions in neurological disorders (Alzheimer's and Parkinson's), inflammation, wound healing, skin diseases, and cancer (33). In addition, RHBDF2 participates in cellular oxidative stress and inflammatory reactions (34).

## Conclusion

EccDNAs and ecDNAs are common in WP and LP. These cDNAs are large enough to carry one or several partial or complete genes. Both WP and LP can potentially be used to construct human biomedical disease models. Besides, there were existed differential abundance of eccDNA (EccDNA00044301) and ecDNA (EcDNA00000060) between both breeds. Future studies should focus on the functional verification of EccDNA00044301 and EcDNA00000060.

## Data availability statement

The data presented in the study are deposited in the China National Center for Bioinformation (CNCB), Genome Sequence Archive (GAS) repository, accession number CRA009400.

## Ethics statement

The animal study was reviewed and approved by Animal Care and Use Committee of Anhui Science and Technology University.

## Author contributions

HH and AW design the study and directed study implementation. WZ managed the fieldwork. HH and WZ performed laboratory work. HH managed data. AW and KW analyzed data. HH, AW, and WZ developed the first draft of the paper. All authors reviewed and edited drafts of the manuscript and approved the final version.
